# Community home elderly care services, multidimensional health and social participation of chronically ill elderly—Empirical analysis based on propensity score matching and multiple mediation analysis

**DOI:** 10.3389/fpubh.2023.1121909

**Published:** 2023-03-23

**Authors:** He Jiang, Zixuan Liu

**Affiliations:** School of Public Adiministration and Law, Hunan Agricultural University, Changsha, China

**Keywords:** community home elderly care services, multidimensional health, chronically ill elderly, social participation, mediation mechanism

## Abstract

**Introduction:**

In recent years, China's aging process has deepened rapidly, the disease spectrum of the population has undergone major changes, the proportion and scale of elderly patients with chronic diseases are growing rapidly, and the multidimensional health problems of the chronically ill elderly are prominent, seriously affecting the participation of the chronically ill elderly in family, community, and social development. In response, China has implemented the “active response to population aging strategy,” accelerated the development of community home elderly care services, and encouraged the chronically ill elderly to continue to play a role through social participation. So how does the community-based home-based medical care service affect the social participation of the chronically ill elderly? Is an important subject.

**Methods:**

Based on the 2018 China Longitudinal Aging Social Survey and the propensity score matching method to measure the impact of community home elderly care services on the four types of social participation of chronically ill elderly, including economic participation, family participation, voluntary participation, and political participation, and group differences, and uses the multiple mediation analysis method to explore the mechanism and effect of community home elderly care services on the four types of social participation of chronically ill elderly.

**Results:**

(1) Community home elderly care services have an asymmetric impact on the social participation of the chronically ill elderly, and have generally promoted the level of social participation of the chronically ill elderly. (2) Community home elderly care services change the social participation arrangements of the chronically ill elderly by driving and inhibiting effects, so that it has a tendency to reduce economic participation and increase voluntary participation and political participation as a whole. This feature shows group differences among the chronically ill elderly of different ages, education levels, living arrangements and community types. (3) Community home elderly care services have an asymmetric impact on various social participation of the chronically ill elderly through the multidimensional health mediation mechanism.

**Conclusion:**

(1) With the continuous deepening of the interweaving between the growth of chronic diseases and the aging of the population in China, the development of a positive aging society must attach great importance to the important role of social participation for the chronically ill elderly. (2) Strengthen the development of physical, psychological, and social adaptation and other health abilities of the chronically ill elderly, shape their awareness of social participation, and give the chronically ill elderly a reasonable social role orientation. (3) Through policy incentives, promote the realization of fairness, justice, adequacy, and sustainability of community home elderly care services. (4) Pay special attention to the penetration, integration, and application of digital technology into the field of community home elderly care services to effectively protect the health of chronically ill elderly, ensure that the elderly with different chronic diseases participate in social activities of high quality, enjoy a high-quality happy life, and promote the high-quality development of the aging society.

## 1. Introduction

According to the data of the 7th National Census, there are 19.064 million people aged 65 and older in China, which accounts for 13.5% of China's total population. It is expected that in 2025 China will have 300 million people over the age of 60, and by mid-century the number will be close to 500 million, accounting for 35% of China's total population, making China a super-aged country under heavy pressure from deep aging. It is worrying that about 76.3% of the elderly in China suffer from chronic diseases, which seriously impair their ability to take care of themselves (1). Because chronic diseases have the characteristics of hidden onset, long course, high cost, and difficulty to cure, they not only damage the health level of the elderly but also greatly increase the pressure of life care and professional medical care for the elderly and reduce their willingness to participate in society. To this end, the whole society has widely gathered the consensus of “active and healthy aging” and believes that Chinese society should continue to establish and improve the elderly care service system “based on home, supported by community, supplemented by institutions, and combined with medical care,” promote “the integrated development of home, community, and institutional elderly care,” promote “the coordination of home, community, and institutions, and the combination of medical and health care,” and community home elderly care services will become a way to achieve the health of all the elderly. It is an important practical way for the whole population to participate and ensure coordinated development in an all-round way (2–4). Therefore, how does the community home elderly care services directly affect the social participation of the chronically ill elderly? How does the community home elderly care services affect the social participation of the chronically ill elderly through multi-dimensional health mediation? This requires a systematic, theoretical answer. For this reason, based on the data of the 2018 China Longitudinal Aging Social Survey, this paper builds a matching regression model for the score of community home elderly care services and a multi-dimensional health mediation model for community home elderly care services and discusses the relationship between “community home elderly care services” and “social participation of the chronically ill elderly.”

## 2. Literature review

### 2.1. Research on community home elderly care services for the chronically ill elderly

With the extension of life span, the incidence rate of chronic diseases in the elderly is getting higher and higher, which requires long-term and sustainable medical services and health management. The separation of medical care and nursing care can not meet the health needs of the elderly. Community home elderly care services have become the focus of the development of China's elderly care service industry. Integrate medical and health resources and endowment service resources, and provide continuous and integrated services such as hospitalization, rehabilitation care, and stable life care for the chronically ill elderly through convenient and professional services (5). For older adults with chronic conditions, family physician services and appropriate medical care services, such as home care and day care, can be provided to older adults, or medical services can be provided through community-based primary care (6). As a kind of integrated care, community home elderly care services integration is a new model of elderly care service worth advocating in China, which is centered on the community, bridging the elderly service centers and health service departments in the community, and integrating rehabilitation and nursing services in the community into an integrated management platform, meeting the wishes of the chronically ill elderly to age at home while taking into account the needs of the chronically ill elderly to age healthily.

### 2.2. Research on the connotation and influencing factors of social participation

Ernest W. Burgess, an American scholar, was the first to introduce the concept of social participation to the elderly population, highlighting the social values that older people have (7). The definition of social participation mainly focuses on the exchange of resources, social relations, and broader social activities. It emphasizes that in the process of social participation, people can contact and interact with others to gain individual value. From the perspective of resource exchange, Bukov et al. (8) believe that social participation is socially oriented individual resource sharing, which can be divided into three types: collective participation, productive participation, and political participation. Levesseur et al. have studied the leisure activities, participation in community activities, and volunteer activities of the elderly (9). Alma et al. (10)has included economic, political, cultural, and other activities in the social participation of the elderly, mainly including economic participation, family participation, voluntary participation, and political participation. Horman and Kiak summarized several decades of relevant research on social participation and finally concluded that the social participation of the elderly is to participate in social activities and interact with people in society and communities (11). Van Hees et al. (12) defined the social participation of older adults as older adults participating in social and productive activities that benefit themselves and society. Secondly, the social participation of the elderly is affected by many factors. Individual characteristics of the elderly, such as age, gender, education, health, marriage, cognitive ability, occupation, social status, political status, economic status, value preference, the social participation concept, and motivation, directly affect the content, mode, and level of social participation of the elderly (13).

### 2.3. Research on health mediation mechanism

Scholars have proposed that care services are conducive to improving the quality of life of the elderly, thus affecting their mental and physical health. When the quality of life of the elderly is low, their physical and mental health will significantly decline, causing significant depression, loneliness, and physical pain (14). Lenardt et al. (15) believed that home-based care can reduce the depression, loneliness, and physical pain of the elderly through regular visits, systematic intervention, and evaluation of the elderly. Zhu et al. (16) proposed the collaboration of community-based elderly institutions and the medical and health care consortium to support the elderly's healthy living abilities at home, allowing the chronically ill elderly to maintain and improve their physical, psychological, and social health. Some scholars also pointed out that care services have a negative impact on the physical and mental health of the elderly. The external stimulation of the elderly receiving services at home will further weaken them, increase their sense of loneliness and isolation, and then present a more inactive state (17). The impact of community home elderly care services on the health of the elderly is determined by the service's quality. When the quality of service meets the needs of the elderly, the physical pain, loneliness, and isolation of the elderly will be weakened, while the service does not meet their own needs, which will cause a series of negative problems such as ADL dysfunction, depression, physical pain perception, life pressure perception, etc. (18). In general, although scholars have different views on the impact of care services on the health of the elderly, they generally agree that community home elderly care services have a significant impact on the health of the elderly. From the perspective of active aging, the social health of the elderly is as important as their mental and physical health. To explore the health of chronically ill elderly, we need to start from multiple dimensions and perspectives, including physical health, mental health, and social health. Secondly, the impact of health status on the social participation of the elderly is particularly concerning: first, physical health, including age and gender. For example, Japanese scholars surveyed 22,845 elderly people's daily instrumental activities and social participation in Nara and other places, and the results confirmed that the daily instrumental activities and social participation of the elderly have a significant correlation and are significantly affected by physical health; South Korean scholars investigated the social participation of 1,346 poor elderly people in South Korea. The results showed that social participation was significantly affected by physical health conditions such as age. The second type of factor is psychological. The significant correlation between mental health and social participation has been confirmed by many studies, especially among older adults, who are at high risk for mental health problems. Depression reduces the likelihood of older adults participating in social or recreational activities (19), seriously affecting their normal lives. Thirdly, social adaptation, i.e., social health, can be seen as the harmony achieved by the interaction between the elderly individual and the social environment and through the elderly's own regulation. The ability of the elderly themselves to adapt socially, i.e., the degree of social health, varies, which affects the process of social participation for the elderly (20).

To sum up, existing studies have discussed the connotation, mode, and determinants of social participation of the elderly in depth and analyzed the impact of health care services on the health of the elderly and the impact of health factors on social participation of the elderly, but the existing literature still has the following deficiencies: research on the impact of multi-focus services on the health of the elderly and the impact of the health of the elderly on social participation. However, it did not further focus on the impact of community home elderly care services on the social participation of the chronically ill elderly, nor did it focus on analyzing whether the community home elderly care services affect the social participation of the chronically ill elderly through multiple health intermediaries. The mediation effect of community home elderly care services and health factors on the social participation of the chronically ill elderly need to be further deepened, expanded, and refined. This paper uses the data of the 2018 China Longitudinal Aging Social Survey and the propensity score matching method to measure the impact of community home elderly care services on the four types of social participation of the chronically ill elderly, including economic participation, family participation, voluntary participation, and political participation, and group differences, and uses the multiple mediation analysis method to explore the mediation effect of community home elderly care services on the four types of social participation of chronically ill elderly.

## 3. Research hypothesis

According to the framework of active aging proposed by the World Health Organization, “health” is the basis of active aging, “participation” is the focus of active aging, and “security” is the necessary condition for the “health” and “participation” of the elderly. The three are organically integrated. From the perspective of participation, the social role theory points out that the social participation of individuals is related to the objective environment or specific situation they face. In order to meet their own needs, the elderly will prioritize various forms of social participation, such as economic participation, political participation, voluntary participation, family participation, and so on, based on the conflict between multiple social role expectations and their limited abilities (21). Maslow's hierarchy of needs theory holds that individual needs are inherently composed of different levels, and the satisfaction of low-level needs is the basic premise for the emergence of high-level needs, which also become the power to encourage and guide individuals to carry out social activities. In other words, the chronically ill elderly also have priorities for various social participation. Only after meeting the most basic needs through social participation can they have the energy to consider high-level social participation. However, the chronically ill elderly are relatively weak in physical function, psychological status and social adaptation, and it is relatively difficult to carry out various social participation. For this problem, social support theory proposes to provide life care, emotional comfort and material support for vulnerable groups, which will help improve the social participation ability of the chronically ill elderly and maximize their sense of participation, sense of gain and sense of happiness. The community home elderly care services mode effectively integrates the resources of medical care and elderly care institutions, not only providing life care, but also providing professional medical care, trying to improve the social participation and quality of life of the chronically ill elderly through service guarantee. It can be seen that community home elderly care services will have an impact on the social participation of the chronically ill elderly. However, the specific impact direction and size will vary with the specific content of social participation. At the same time, the chronically ill elderly with different age, education level, living arrangement, community type and other characteristics, after using community home elderly care services, they may make differentiated social participation arrangements because of their own social participation capacity differences or differences in various social participation needs. Therefore, the following assumptions are proposed:

H1: Community home elderly care services have an asymmetric impact on the economic participation, political participation, voluntary participation, and family participation of the chronically ill elderly, and there are group differences among the chronically ill elderly with different characteristics.

Community home elderly care services model aims at healthy aging for the whole society and aims at maintaining the functions of the elderly, promoting their social participation, bringing out their values, and improving their wellbeing, thus enhance the health of the elderly (22). Health status in turn affects the level of social participation of older adults. The physical health status of chronically ill elderly adults is the primary factor limiting their social participation. In addition to being related to physical health, social participation is also related to mental health and other factors. Psychological factors such as emotions can affect a person's ability to be active. Excessive stress or extreme depression can cause mood swings that affect their activities, such as a reluctance to approach people when sad, depressed, or bored and reduced social participation. Social health, on the other hand, refers to the individual's active interaction with social relationships in social roles and the various feelings of gain and identity gained from social activities. Unlike physical and mental health, social health is more interactive, and a person with a high level of social health should be actively involved in social activities. However, the processes and mechanisms by which community home elderly care services affect the social participation of chronically ill elderly people through their health status are currently unclear. However, considering the asymmetric impact of community home elderly care services on the health of the elderly and the asymmetric impact of health on the social participation of the elderly, it can be theoretically concluded that the mediation role of community home elderly care services in influencing the social participation of the chronically ill elderly through physical health, mental health, and social health is asymmetric. Therefore, the following hypothesis is proposed:

H2: Community home elderly care services influence social participation asymmetrically through the mediating effects of physical health, mental health, and social health.

**Figure 1:**
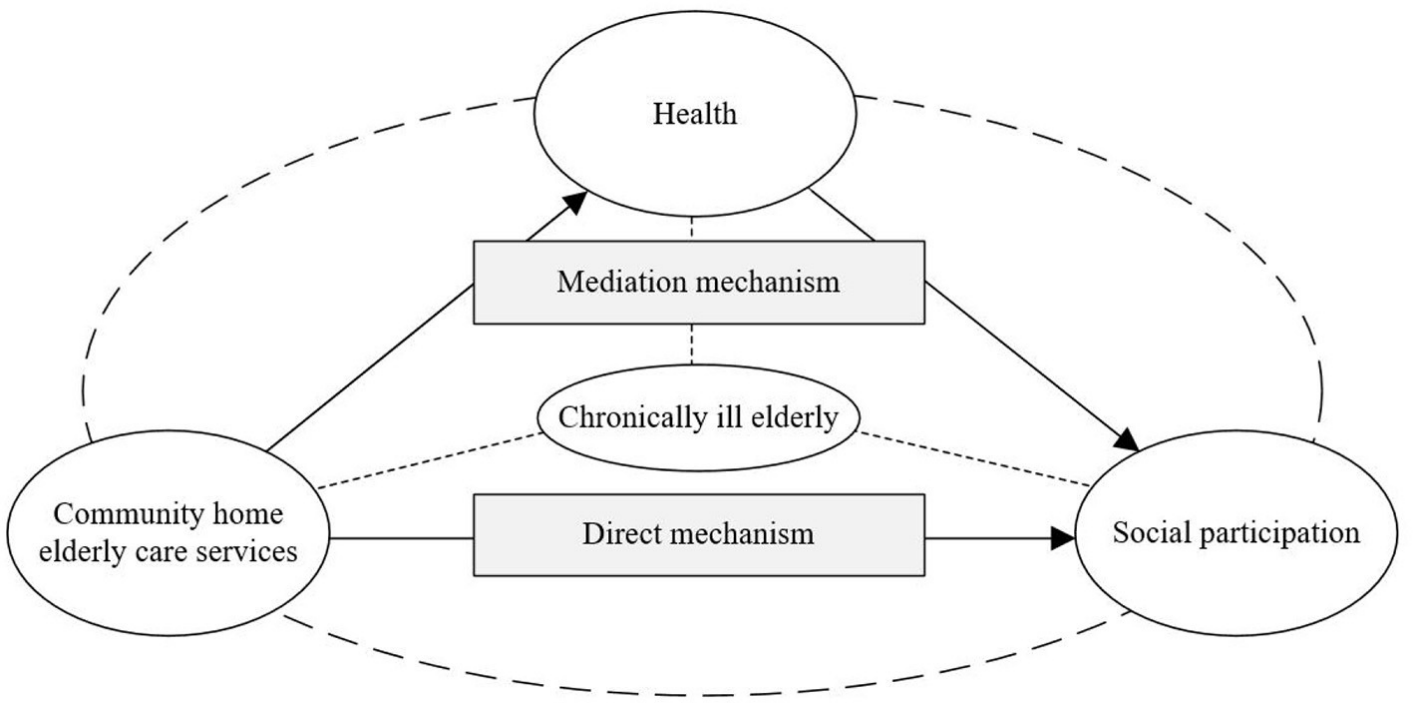
Analysis framework: influence mechanisms between community home elderly care services, health status, and social participation of chronically ill elderly.

Based on the research hypothesis, this paper constructs a mechanism analysis framework for exploring community-based home health care services, multidimensional health, and social participation of the chronically ill elderly, as shown in Figure 1.

## 4. Research design

### 4.1. Data sources

The data in this article is based on the 2018 China Longitudinal Aging Social Survey (CLASS). The survey adopted a multi-level and multi-stage probability sampling method. Eleven thousand four hundred nineteen samples were collected from 462 villages (residents) in 28 provinces, autonomous regions, and municipalities (except Hainan, Xinjiang, Tibet, Hong Kong, Macao, and Taiwan), covering information about the health, social participation, and community environment of the elderly aged 60 and above. As this paper focuses on the chronically ill elderly, a sample of chronically ill elderly was analyzed. After variable screening and missing value processing, 5,555 valid samples were finally obtained.

### 4.2. Variable description

#### 4.2.1. Dependent variable

This paper's dependent variable is social participation. The types and contents of social participation are very rich. Based on the literature, this paper measures the social participation of the chronically ill elderly in four dimensions: economic participation, family participation, voluntary participation, and political participation (23, 24).

First, economic participation is a binary variable set to “participation = 1, non-participation = 0” in the questionnaire item “Current Situation of Your Work/Activity with Income.”

Second, family participation is set as a binary variable of “participation = 1, non-participation = 0” according to parental care, household help, and intergenerational care of the chronically ill elderly. The term “parental care” is derived from the question, “Do your own (or your spouse's) parents still require care?” Household help comes from the question, “How often have you helped this child do housework in the past 12 months?” The intergenerational care is derived from the question item, “The time you spent taking care of your first child in the past 12 months.”

Third, according to the questionnaire item, “How often have you participated in the following activities in the past year?” Set as a binary variable of “participation = 1, non-participation = 0.” These voluntary activities include community security patrols, caring for other elderly or children, environmental health protection, neighborhood dispute mediation, accompanying chats, voluntary services, caring for and educating the next generation, etc.

Fourth, political participation is based on the questionnaire item, “Have you participated in the voting of the local residents' committee or villagers' committee in the past 3 years?” Generate a binary variable of “participation = 1, non-participation = 0.” Table 1 shows that the social participation rates of the chronically ill elderly in economy, family, volunteering, and politics are 22.7%, 54.3%, 31.4%, and 40.9%, respectively.

**Table 1:** Descriptive statistics of variables.

**Variable**	**Variable type**	**Assignment description**		**Full sample**		**Treatment group**		**Control group**	
				**Mean value**	**SD**	**Mean value**	**SD**	**Mean value**	**SD**
**Dependent variable**									
Economic participation	Dummy	Participation = 1, non-participation = 0		0.227	0.419	0.188	0.390	0.253	0.435
Family participation	Dummy	Participation = 1, non-participation = 0		0.543	0.498	0.539	0.499	0.546	0.498
Voluntary participation	Dummy	Participation = 1, non-participation = 0		0.314	0.464	0.350	0.477	0.290	0.454
Political participation	Dummy	Participation = 1, non-participation = 0		0.409	0.492	0.446	0.497	0.385	0.487
**Independent variable**									
Community home elderly care services	Dummy	Have used any community home elderly care services, the answer is 1, otherwise it is 0		0.393	0.488	\	\	\	\
**Mechanism variable**									
Physical health	Category	Score between 12–34		33.059	2.456	32.835	2.879	33.205	2.125
Mental health	Continuous	Score between 12–36		27.647	4.107	27.982	4.081	27.430	4.110
Social health	Continuous	Score between 8–24		16.421	3.104	16.619	3.815	16.307	3.178
**Control variable**									
Personal characteristics	Age	Continuous	Calculated according to the date of birth	71.434	7.189	71.996	7.448	71.070	6.993
	Gender	Dummy	Male = 1, female = 0	0.497	0.500	0.477	0.500	0.510	0.500
	Registered residence	Dummy	Non-agricultural household registration = 1, otherwise = 0	0.516	0.500	0.561	0.496	0.488	0.500
	Education level	Category	Below primary school = 0, primary school = 1, junior high school = 2, senior high school and above = 3	1.212	0.974	1.161	0.966	1.245	0.978
	Digital literacy	Dummy	Being able to engage in chatting, shopping, browsing information, entertainment, transportation, health management, investment and financing, and learning and training through a digital network = 1, otherwise = 0	0.214	0.410	0.217	0.412	0.213	0.409
	Major event experience	Dummy	Whether you have experienced important events in the past 12 months	0.282	0.450	0.333	0.471	0.249	0.432
	Smoking	Dummy	Still smoking = 1, no longer smoking = 0, never smoked = 0	0.215	0.411	0.222	0.416	0.210	0.407
	Number of chronic diseases	Continuous	According to the number of chronic diseases	11.221	7.613	11.148	7.635	11.268	7.599
Family characteristics	Marriage	Dummy	Married = 1, widowed, divorced, unmarried = 0	0.683	0.465	0.669	0.471	0.692	0.462
	Living alone	Dummy	Living alone = 1, otherwise = 0	0.878	0.328	0.871	0.335	0.882	0.323
	Number of children	Dummy	Number of living children	2.505	1.344	2.386	1.323	2.581	1.353
	Family income	Continuous	Logarithm of per capita household income	8.147	1.401	8.289	1.369	8.055	1.414
Social characteristics	Endowment insurance	Dummy	Have enjoyed the basic endowment insurance of enterprise employees, government institutions and urban and rural residents = 1, otherwise = 0	0.801	0.399	0.885	0.319	0.746	0.435
	Type of residential community	Dummy	Living in rural community = 1, otherwise = 0	0.398	0.489	0.360	0.480	0.422	0.494
Sample size				5,555		2,180		3,375	

#### 4.2.2. Independent variable

The independent variable of this article is the community home elderly care services. This variable is based on whether the respondents in the questionnaire have used such items as “home visit, home care, home visit, home housework, elderly service hotline, accompanying doctor, helping daily shopping, legal aid, elderly dining table or delivery, day care station or nursing home, psychological consultation, rehabilitation training, rental of rehabilitation aids, free physical examination, establishment of health files, health lectures,” a binary variable set to “any service used = 1, no service used = 0 (25).” Table 1 shows that in 2018, about 39% of the chronically ill elderly were covered by community home elderly care services in China.

#### 4.2.3. Mechanism variables

According to the literature and research assumptions, this paper focuses on the impact of community home elderly care services on the social participation of the chronically ill elderly through the three mechanism variables of physical health, mental health, and social health (26).

First, the physical health was measured objectively by the “Activity of Daily Living (ADL)” scale consisting of 12 items in the questionnaire, which include the basic activity of daily living (BADL) and instrumental activity of daily living (IADL). Among them, BADL includes dressing, bathing, eating, defecation control, toileting, indoor action, and other items, while IADL includes bus travel, shopping, money management, lifting 10 kg, cooking, housework, and other items. In this paper, the answer items “don't need help from others,” “need some help,” and “can't do it at all” are assigned three points, two points, and one point, respectively, and the answer items “can” and “can't” are assigned two points and one point, respectively, and the continuous variable between 12 and 34 is obtained by summing up. The higher the value, the better the physical health. The Cronbach's alpha reliability coefficient test of the sample is 0.897, indicating that the scale has high reliability.

Secondly, mental health was investigated according to the “Depression” scale, consisting of 12 items in the questionnaire that reflected the respondents' psychological states of “mood, loneliness, sadness, life satisfaction, diet, sleep, interest, companionship, feelings of not being useful, feelings of having nothing to do, feelings of being ignored, and feelings of being isolated.” The answers of “no,” “sometimes,” and “often” were assigned one point, two points, and three points, respectively, and the negative items were summed up after being processed in reverse order, A continuous variable between 12 and 36 is obtained. The higher the value, the lower the depression and mental health of the chronically ill elderly. The Cronbach's reliability coefficient is 0.758, which indicates that the reliability of the scale is high.

Third, social health was investigated according to the eight items in the questionnaire that reflected the respondents' “enthuasia for work participation, social dedication, love for learning, self-social value perception, social change adaptation, acceptance of social concepts, acceptance of social policies, and awareness of social friendliness.” The answers “completely inconsistent” and “relatively inconsistent” were assigned one point each, “general” was assigned two points, the values of “relatively consistent” and “completely consistent” were assigned three points, and the negative items were processed in reverse order and summed up to obtain continuous variables with values between 8 and 24. The higher the score, the better the social health. The Cronbach's alpha reliability coefficient is 0.826, which meets the data quality requirements.

#### 4.2.4. Control variables

According to the existing literature, this paper controls the individual, family, and social characteristics of the chronically ill elderly to reduce the research bias caused by the omission of variables (27, 28). Among them, individual characteristics include age, gender, registered residence, education level, digital literacy, major events experience, smoking, and number of chronic diseases; family characteristics include marriage, living alone, number of children, and family income; social characteristics include variables such as endowment insurance and type of residential community.

The specific variable meaning, assignment, and descriptive analysis results are shown in Table 1.

### 4.3. Research methods

#### 4.3.1. Propensity score matching

In the study of inferring the actual causal relationship between two variables, selective bias and mixed bias often seriously interfere with the estimation results. The ideal test is to adopt the random test method of completely controlling the characteristic variables (29). Therefore, the best method in this study is to reveal the impact of community home elderly care services on the social participation of the chronically ill elderly by comparing the difference in social participation between the treatment group (the chronically ill elderly who use community home elderly care services) and those who do not receive community home elderly care services. However, in reality, it is impossible to observe whether the social participation of the treatment group will change without using community home elderly care services, because this is a “counterfactual.” To solve this problem, Rosenbaum and Rubin (30) proposed a counterfactual inference model of the propensity score matching method (PSM). Its basic idea is to build a counterfactual framework that can approximate randomizing non-random data by finding counterfactual control groups similar to the processing group while simultaneously reducing the dimension of multiple observable characteristic variables to match, so as to eliminate sample bias to the maximum extent and ensure more accurate estimation results. The specific ideas are as follows:

The first step is to use the Logistic model to estimate the tendency score of the chronically ill elderly to use community home elderly care services:

(1) $$\begin{array}{l} Logit\left(Service_{i}=1\right)=\alpha +\beta X_{i}+e_{i} \end{array}$$

Among them, *Service*_*i*_ is the community home elderly care services, and *X*_*i*_ is the vector composed of personal, family, and social characteristic variables.

The second step is to find comparable objects with similar propensity scores for each treatment group sample in the control group for pair analysis and ensure effective matching by jointly supporting the hypothesis test and balance test. At present, the academic community has not reached a consensus on the selection of specific matching methods. In this paper, the nearest neighbor matching method is selected first, and other matching methods are replaced in the robustness test.

The third step is to estimate the average treatment effect for the treated (ATT) of the treatment group according to the matched samples:

(2) $$\begin{array}{l} ATT=E\left\{E\left[Y_{1i}-Y_{0i}|D_{i}=1\right]\right\}=E\left\{E\left[Y_{1i}|D_{i}=1\right]-E\left[Y_{0i}|D_{i}=0\right]\right\} \end{array}$$

Among them, *Y*_1*i*_ and *Y*_0*i*_ represent the social participation indicators of the chronically ill elderly who use and do not use community home elderly care services respectively; *D*_*i*_ is a key independent variable, indicating whether the chronically ill elderly use community home elderly care services. If they have, *D*_*i*_ = 1; otherwise, *D*_*i*_ = 0.

#### 4.3.2. Multiple mediation analysis

Since the introduction of the causality stepwise regression test by Baron and Kenny (31), the mediation analysis method has evolved rapidly and gradually from simple mediation analysis of a single mediation variable to multiple mediation analysis of multiple mediation variables. At the same time, more attention has been paid to clarifying the three mechanisms of mediation effect, confounding effect, and suppressing effect in mediation analysis. Both mediation effect variables and confounding effect variables can reduce the total effect of independent variables on dependent variables. The difference is that mediation effect variables are in the causal chain between independent variables and dependent variables, while confounding effect variables are not necessarily in the causal relationship between them; the suppressing effect will increase the total effect of independent variables on dependent variables (32). This paper intends to use multiple mediation analysis to reveal the mechanism of community home elderly care services affecting the social participation of the chronically ill elderly through three mediation variables: physical health, mental health, and social health. The relationship function between the main variables in the multiple mediation analysis is:

(3) $$\begin{array}{r} Y_{ip}=\beta_{0}^{1}+\beta_{j}^{1}Service_{ij}+\overset{n}{\underset{l=1}{\sum }}\beta_{l}^{1}X_{il}+\varepsilon_{i}^{1} \end{array}$$

(4) $$\begin{array}{r} Health_{ik}=\beta_{0}^{2}+\beta_{j}^{2}Service_{ij}+\overset{n}{\underset{l=1}{\sum }}\beta_{l}^{2}X_{il}+\varepsilon_{i}^{2} \end{array}$$

(5) $$\begin{array}{r} Y_{ip}=\beta_{0}^{3}+\beta_{j}^{3}Service_{ij}+\overset{3}{\underset{k=1}{\sum }}\beta_{k}^{3}Health_{ik}+\overset{n}{\underset{l=1}{\sum }}\beta_{l}^{3}X_{il}+\varepsilon_{i}^{3} \end{array}$$

Among them, *Y*_*ip*_(*p* = 1, 2, 3, 4) represents the four dependent variables of economic participation, family participation, voluntary participation, and political participation of the chronically ill elderly; *Health*_*ik*_(*k* = 1, 2, 3) represents the three mediation variables of physical health, mental health, and social health; and ε represents the error term. Formula (3) is used to test the direct effect of community home elderly care services on the social participation of the chronically ill elderly; Formula (4) is used to test the impact of community home elderly care services on health mediation variables; the indirect effect of mediation variables can be obtained by substituting Formula (4) for Formula (5). If coefficients $\beta_{j}^{2}$ and $\beta_{k}^{3}$ are significant, it can be determined that community home elderly care services affect the social participation of the chronically ill elderly through health mediation variables.

## 5. Empirical results

This part uses the propensity score matching model to analyze the direct effects of community home elderly care services on the economic participation, family participation, voluntary participation, and political participation of the chronically ill elderly and discusses the group differences of community home elderly care services on the social participation of the elderly with different types of chronic illnesses.

### 5.1. Propensity score matching quality analysis

According to formula (1) above, calculate the propensity score of the chronically ill elderly to receive community home elderly care services and use the K nearest neighbor matching (*K* = 1, caliper = 0.05) method to match the propensity scores of various characteristics of the treatment group and the control group, and then test the common support hypothesis and balance hypothesis to ensure that the PSM estimation is effective.

**Figure 2:**
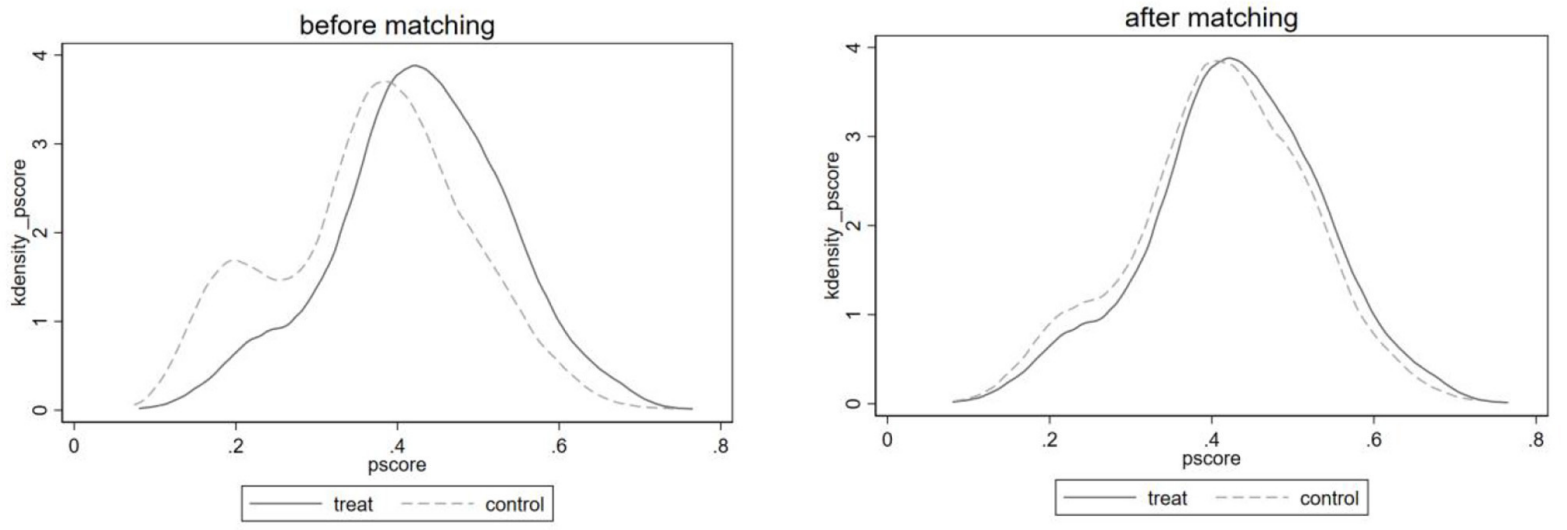
Nuclear density distribution of propensity scores of treatment group and control group before and after matching.

First of all, the common support hypothesis requires that the trend values of the treatment group and the control group have a common range of values. As shown in Figure 2, there is a significant difference in the nuclear density distribution between the pre-matching treatment group and the control group. After matching, the trend of the nuclear density curves of the two groups of samples tends to be consistent and highly fitted, indicating that the matching effect is ideal and meets the common support hypothesis.

Secondly, the PSM balance hypothesis requires that there be no significant difference in control variables between the treatment group and the control group after sample matching (Table 2). Balance test results show that the standardized deviation of each control variable in the sample matching post-processing group and the control group is greatly reduced, and their absolute values are less than the 20% red line specified in the balance test, which indicates that the selection of matching variables is more reasonable, meets the balance assumption, and the matching quality is high, which can effectively reduce the estimation error caused by sample self-selection.

**Table 2:** Balance test results.

**Variable**	**Logit Model**	**Matching status**	**Mean**		**Mean difference test**	
			**Treated**	**Control**	**Bias (%)**	***T*****-test** ***p*** > **|*****t*****|**
Age	0.026^***^	Unmatched	72.006	71.070	13.0	0.000
	(0.005)	Matched	71.990	72.038	−0.7	0.829
Gender	−0.142^**^	Unmatched	0.477	0.510	−6.6	0.016
	(0.064)	Matched	0.477	0.501	−4.7	0.122
Registered residence	0.047	Unmatched	0.561	0.488	14.7	0.000
	(0.095)	Matched	0.561	0.554	1.3	0.669
Education level	−0.212^***^	Unmatched	1.162	1.245	−8.5	0.002
	(0.036)	Matched	1.163	1.202	−4.0	0.188
Digital literacy	0.023	Unmatched	0.217	0.213	0.9	0.738
	(0.080)	Matched	0.217	0.219	−0.7	0.826
Major event experience	0.432^***^	Unmatched	0.333	0.249	18.6	0.000
	(0.063)	Matched	0.332	0.348	−3.5	0.277
Smoking	0.157^**^	Unmatched	0.222	0.210	2.9	0.290
	(0.075)	Matched	0.222	0.226	−1.0	0.744
Number of chronic diseases	−0.007^*^	Unmatched	11.148	11.268	−1.6	0.567
	(0.004)	Matched	11.147	11.003	1.9	0.528
Marriage	0.053	Unmatched	0.669	0.692	−5.0	0.068
	(0.075)	Matched	0.669	0.667	0.8	0.797
Living alone	−0.103	Unmatched	0.871	0.882	−3.2	0.236
	(0.101)	Matched	0.872	0.862	2.9	0.349
Number of children	−0.175^***^	Unmatched	2.386	2.581	−14.6	0.000
	(0.025)	Matched	2.388	2.392	−0.3	0.925
Family income	0.080^***^	Unmatched	8.289	8.055	16.7	0.000
	(0.025)	Matched	8.289	8.301	−0.9	0.780
Endowment insurance	0.950^***^	Unmatched	0.885	0.746	36.3	0.000
	(0.083)	Matched	0.885	0.887	−0.5	0.849
Type of residential community	0.070	Unmatched	0.360	0.422	−12.6	0.000
	(0.094)	Matched	0.360	0.350	2.1	0.486

*** p < 0.01,

** p < 0.05,

* p < 0.1, respectively indicate that the estimated results are significant at 1%, 5%, and 10% levels. The parentheses are standard errors.

### 5.2. Propensity score treatment effect analysis

Table 3 reports the average treatment effect for the treated (ATT) of the use of community home elderly care services on the economic participation, family participation, voluntary participation, and political participation of the chronically ill elderly under the four methods of *K*-nearest neighbor matching, radius matching, nuclear matching, and Markov matching, after controlling the characteristic variables. The results showed that there was no significant difference between the ATT and t values obtained by the four matching methods, indicating that the influence of community home elderly care services on the social participation of the chronically ill elderly was not interfered with by the propensity score matching method and had good robustness. The arithmetic mean value of ATT of four matching methods was selected for further analysis, and it was found that community home elderly care services had an asymmetric impact on the social participation of the chronically ill elderly. First, in terms of economic participation, the treatment group's average treatment effect was −0.032, implying that using community home elderly care services significantly reduced the economic participation rate of the chronically ill elderly by 3.2%; Second, the average treatment effect of the treatment group in terms of voluntary participation and political participation was 0.049 and 0.067, respectively, indicating that the use of community home elderly care services significantly increased the voluntary participation rate of 4.9% and the political participation rate of 6.7% of the chronically ill elderly; Finally, in terms of family participation, the evaluation and treatment effect of the treatment group is not significant, that is, the use of community home elderly care services has a neutral effect on family participation of the chronically ill elderly.

**Table 3:** Average treatment effect for the treated of different matching methods.

**Matching method**	**Economic participation**		**Family participation**		**Voluntary participation**		**Political participation**	
	**ATT**	***T*** **-value**	**ATT**	***T*** **-value**	**ATT**	***T*** **-value**	**ATT**	***T*** **-value**
K-nearest neighbor matching(K=1)	−0.036^**^ (0.016)	−2.25	0.024 (0.019)	1.22	0.046^*^ (0.018)	2.56	0.079^**^ (0.019)	4.17
Radius matching (0.001)	−0.031^**^ (0.012)	−2.53	0.014 (0.015)	0.96	0.050^***^ (0.014)	3.56	0.069^***^ (0.015)	4.066
Nuclear matching	−0.027^**^ (0.012)	−2.34	0.005 (0.014)	0.32	0.043^***^ (0.013)	3.20	0.058^***^ (0.014)	4.07
Markov matching	−0.032^**^ (0.011)	−3.01	−0.009 (0.015)	−0.59	0.056^***^ (0.014)	4.08	0.059^***^ (0.015)	4.300
ATT average	−0.032		0.009		0.049		0.067	

*** p < 0.01,

** p < 0.05,

* p < 0.1, respectively indicate that the estimated results are significant at 1%, 5%, and 10% levels. The parentheses are standard errors.

It can be seen that the use of community home elderly care services will change the social participation arrangements of the chronically ill elderly and make them show a tendency to reduce economic participation and increase voluntary participation and political participation. On the one hand, this is because community home elderly care services help improve the quality of life of the chronically ill elderly, improve the demand level of the chronically ill elderly, enhance the social participation ability of the chronically ill elderly, and ultimately reduce the economic participation of the chronically ill elderly and promote their voluntary participation and political participation. On the other hand, it can be explained that the chronically ill elderly will adjust their social role response strategies after using community home elderly care services according to their social roles and the differentiated functions of the four types of social participation and form a new balance of social participation arrangements. On the other hand, the chronically ill elderly under the Chinese “family” culture have a profound “father is kind and son is filial” blood relationship and the filial piety gene. Because of the rigid value preferences of the chronically ill elderly for filial piety, ancestor worship, inheritance, and blood continuity, community home elderly care services will find it difficult to influence family participation.

### 5.3. Group difference analysis

The chronically ill elderly will be affected differently by community home elderly care services due to their different age stages, education levels, residential arrangements, and community-types. Table 3 reports the average effect of community home elderly care services on the social participation of the chronically ill elderly, but it cannot reflect the structural difference in the impact effect of the chronically ill elderly, that is, group differences. This paper discusses the group differences of different types of elderly people, which will help enrich the research content of community home elderly care services on the social participation of the chronically ill elderly. The group difference results based on the nearest neighbor matching method are shown in Table 4.

**Table 4:** Group differences in the impact of community home elderly care services on the social participation (ATT) of the chronically ill elderly.

**Variable**		**Variable sample size**	**Economic participation**		**Family participation**		**Voluntary participation**		**Political participation**	
			**ATT**	***T*** **-value**	**ATT**	***T*** **-value**	**ATT**	***T*** **-value**	**ATT**	***T*** **-value**
Age	60–69 years old	2662	0.004 (0.025)	0.16	−0.009 (0.026)	−0.33	0.059^**^ (0.026)	2.23	0.089^***^ (0.028)	3.17
	70–79 years old	2017	−0.029 (0.025)	−1.19	0.017 (0.033)	0.55	0.045 (0.030)	1.49	0.083^***^ (0.032)	2.64
	80 years old and above	876	−0.104^***^ (0.034)	−3.12	−0.078^*^ (0.047)	−1.65	−0.002 (0.043)	−0.06	0.077^*^ (0.044)	1.74
Education level	Primary school and below,	3521	−0.037^*^ (0.022)	−1.72	−0.005 (0.025)	−0.20	0.112^***^ (0.021)	5.28	0.020 (0.025)	0.08
	Junior high school	1377	−0.035 (0.028)	−1.28	−0.015 (0.037)	−0.42	−0.049^*^ (0.037)	−1.33	0.142^***^ (0.037)	3.86
	High school and above	657	0.026 (0.035)	0.74	−0.065 (0.057)	−0.11	0.116^**^ (0.058)	2.00	0.142^**^ (0.057)	2.49
Residential arrangement	Living alone	680	−0.028^*^ (0.017)	−1.65	0.010 (0.020)	0.49	0.027^**^ (0.019)	1.44	0.073^***^ (0.019)	3.67
	No living alone	4875	−0.004 (0.045)	−0.08	−0.022 (0.052)	−0.42	0.115^**^ (0.051)	2.27	0.093^*^ (0.053)	1.76
Community-type	Non-agricultural community	3347	−0.004 (0.012)	−0.36	0.017 (0.024)	0.70	0.043^*^ (0.023)	1.84	0.073^***^ (0.023)	3.13
	Rural communities	2208	−0.048 (0.031)	−1.52	−0.012 (0.031)	−0.37	0.002^**^ (0.028)	−0.07	0.045 (0.032)	1.41

^***^, ^**^, and ^*^ indicate significance at 1%, 5%, and 10% levels. The parentheses are standard errors.

#### 5.3.1. Age group differences

With reference to the age standard of the United Nations, this paper divides the samples into three groups: the young elderly (60–69 years old), the middle-aged elderly (70–79 years old), and the elderly (80 years old and above) to explore the potential differences in the impact of community home elderly care services on the chronically ill elderly at different ages. It can be seen from Table 4 that the community home elderly care services have a significant positive impact on the voluntary participation and political participation of the young people aged 60–69, a significant negative impact on the political participation of the middle-aged people aged 70–79, a significant negative impact on the economic participation and family participation of the elderly aged 80 and above, and a significant positive impact on their political participation.

#### 5.3.2. Educational group differences

According to the education level of the chronically ill elderly, this paper is divided into three groups: primary school and below, junior high school, senior high school and above. It can be seen from Table 4 that the community home elderly care services have a significant negative impact on the economic participation of the chronically ill elderly at primary school and below and a significant positive impact on voluntary participation; the community home elderly care services have a significant negative impact on the voluntary participation of the chronically ill elderly at junior high school level and a significant positive impact on political participation; and the community home elderly care services have a significant positive impact on the voluntary participation and political participation of the chronically ill elderly at high school and above.

#### 5.3.3. Residential arrangement group differences

According to the residential arrangements of the chronically ill elderly, this paper divides them into two groups: solitary and non-solitary. It can be seen from Table 4 that the community home elderly care services have a significant positive effect on the voluntary participation and political participation of the chronically ill elderly living alone and not living alone, but the effect on the chronically ill elderly not living alone is greater than that of the chronically ill elderly living alone. In addition, community home elderly care services have a significant negative impact on the economic participation of the elderly living alone with chronic diseases.

#### 5.3.4. Community-type group differences

For analysis, this paper divides the types of residential communities for the chronically ill elderly into two groups: non-agricultural communities and rural communities. It can be seen from Table 4 that the community home elderly care services have a significant positive impact on the voluntary participation and economic participation of the chronically ill elderly living in non-agricultural communities, but only has a significant positive impact on the voluntary participation of the chronically ill elderly in rural communities.

## 6. Mechanism analysis

The above research focuses on how community home elderly care services can affect the social participation of the chronically ill elderly. In order to further clarify how community home elderly care services affect the social participation of the chronically ill elderly, this paper uses the bias-corrected non-parametric Bootstrap method to conduct a multiple mediation analysis of 5,000 repeated samples to test the mechanism by which the community home elderly care services affect the social participation of the chronically ill elderly.

Table 5 shows that in the path of community home elderly care services affecting the economic participation of the chronically ill elderly, the total direct effect is significantly negative at the 1% statistical level, the mediation effects of physical health and mental health are significantly negative, and the mediation effect of social health is significantly positive, indicating that physical health, mental health, and social health play a “partial mediation effect.” In other words, community home elderly care services not only have a direct negative impact on the economic participation of the chronically ill elderly but also inhibit the economic participation of the chronically ill elderly through the negative “mediation effect” of physical health and mental health, which account for 15.5 and 18.3% of the total effect, respectively, and also promote the economic participation of the chronically ill elderly through the positive “mediation effect” of social health, which accounts for 4.6% of the total effect.

**Table 5:** Multiple health mediating effects.

**Impact path**	**Total direct effect**	**Mediation effect**	**95% BootLLCI**	**95% BootULCI**	**Proportion of mediation effect or suppressing effect value (%)**
1.Community home elderly care services → physical health → economic participation	−0.268^***^ (0.085)	−0.051 (0.017)	−0.091	−0.021	15.5%
2.Community home elderly care services → mental health → economic participation		−0.060 (0.019)	0.006	0.029	18.3%
3.Community home elderly care services → social health → economic participation		0.015 (0.006)	−0.103	−0.025	4.6%
4.Community home elderly care services → physical health → family participation	0.059 (0.061)	−0.015 (0.006)	−0.028	−0.005	44.4%
5.Community home elderly care services → mental health → family participation		−0.003 (0.005)	−0.014	0.007	[5.1%]
6.Community home elderly care services → social health → family participation		−0.007 (0.004)	−0.016	−0.001	20.7%
7.Community home elderly care services → physical health → voluntary participation	0.278^***^ (0.064)	0.0002 (0.003)	−0.006	0.006	[0.1%]
8.Community home elderly care services → mental health → voluntary participation		0.043 (0.009)	0.027	0.062	13.3%
9.Community home elderly care services → social health → voluntary participation		0.019 (0.004)	−0.005	0.010	[6.8%]
10.Community home elderly care services → physical health → political participation	0.268^***^ (0.060)	−0.018 (0.066)	−0.033	−0.007	6.6%
11.Community home elderly care services → mental health → political participation		0.008 (0.005)	−0.002	0.019	[2.9%]
12.Community home elderly care services → social health → political participation		0.013 (0.005)	0.006	0.024	4.8%

*** p < 0.01,

** p < 0.05,

* p < 0.1, respectively indicate that the estimated results are significant at 1%, 5%, and 10% levels. The parentheses are standard errors. The value in brackets is the suppressing effect value, which is calculated by dividing the suppressing effect by the direct effect (taking the absolute value). The percentage of mediation effect is the percentage of mediation effect in total effect. If the BootLLCI–BootULCL interval does not contain 0, the indirect effect is significant; the estimation results of control variables are omitted.

In the path of community home elderly care services affecting the family participation of the chronically ill elderly, the total direct effect is positive but not significant, the mediation effect of physical health and social health is significantly negative, and the mediation effect of mental health is not significant, indicating that the community home elderly care services inhibit the family participation of the chronically ill elderly through the negative “complete mediation effect” of physical health and social health, which account for 44.4% and 20.7% of the total effect, respectively. Mental health has a “suppressing effect.”

In the path of community home elderly care services affecting the voluntary participation of the chronically ill elderly, the total direct effect is significantly positive at the 1% statistical level, the mediation effect of mental health is significantly positive, and the mediation effect of physical health and social health is not significant, indicating that community home elderly care services promote the political participation of the chronically ill elderly through the positive “partial mediation effect” of mental health, which accounts for 13.3% of the total effect. Physical and social health both have a “suppressing effect.”

The total direct effect of community home elderly care services influencing the political participation of the chronically ill elderly is significantly positive at the 1% statistical level; the indirect effect of physical health is significantly positive; the indirect effect of social health is significantly negative; and the indirect effect of mental health is not significant, indicating that the community home elderly care services affect the political participation of the chronically ill elderly through the positive “partial mediation effect” of physical health and the negative “partial mediation effect” of social health, which account for 6.6 and 4.8% of the total effect, respectively. Mental health has a “suppressing effect.”

Based on the above results, it can be seen that community home elderly care services affect the social participation of the chronically ill elderly through the asymmetry of direct mechanisms and mediation mechanisms. In terms of direct mechanisms, community home elderly care services negatively drive economic participation and positively drive voluntary participation and political participation. In terms of mediation mechanisms, community home elderly care services negatively affect economic participation, family participation, and political participation through the physical health mediation mechanism, negatively affect economic participation and positively drive voluntary participation through the psychological health mediation mechanism, and positively drive economic participation and political participation and negatively drive family participation through the social health mediation mechanism. Hypotheses 1 and 2 are verified.

## 7. Conclusion and discussion

Research findings: (1) The community home elderly care services have an asymmetric impact on the social participation of the chronically ill elderly. It has significantly reduced the economic participation rate of the chronically ill elderly by 3.2%, significantly increased the voluntary participation rate of the chronically ill elderly by 5.6%, and increased the political participation rate by 5.9%, but has not significantly affected the family participation of the chronically ill elderly. It has generally promoted the social participation of the chronically ill and elderly. (2) At the same time, community home elderly care services change the social participation arrangements of the chronically ill elderly by driving and inhibiting effects, so that they have a tendency to reduce economic participation and increase voluntary participation and political participation as a whole. This feature shows group differences among the chronically ill elderly of different ages, education levels, living arrangements, and community types. (3) Community home elderly care services have an asymmetric impact on various social participations of the chronically ill elderly through the multidimensional health mediation mechanism: a negative impact on economic participation, family participation, and political participation through the physical health mediation mechanism; a negative impact on economic participation and a positive drive on voluntary participation through the psychological health mediation mechanism; a positive drive on economic participation and political participation and a negative drive on family participation through the social health mediation mechanism.

Among the three pillars of “health,” “participation,” and “security,” the framework of active aging emphasizes the importance of “participation,” emphasizes that it is based on security and supported by health, and encourages the elderly to actively engage in social participation activities. The chronically ill elderly are vulnerable groups with poor physical function, mental state, and social adaptability. Their basic ability to reasonably arrange social participation in the face of social role conflict is lower, and their social participation still has a large room for adjustment in realizing their old age and meeting social expectations (33). At this time, it is necessary to carry out security interventions for the chronically ill elderly (34). This study shows that at present, the social participation of the chronically ill elderly in China shows the basic characteristics of “family participation dominating, followed by political participation and voluntary participation, and economic participation being the least.” However, the community home elderly care services obviously adjust the social participation arrangements of the chronically ill elderly, reduce their economic participation, and increase their social participation in voluntary and political fields, while family participation has not changed significantly. In the dimension of economic participation, the human capital, motivation for participation, and employment opportunities of the elderly are the main factors that determine their income activities (35). The chronically ill elderly have poor physical function, psychological status, and social adaptability, and the level of human capital is relatively low, so the competitiveness of economic participation is relatively weak, making their employment level often low. In particular, as a security model focusing on family and community fields to provide health care services for the elderly, the community home elderly care services may, on the one hand, weaken their motivation for economic participation by alleviating the life pressure of the chronically ill elderly and improving their quality of life, and, on the other hand, lock the social space of the chronically ill elderly in the scope of family and community, thus reducing their chances of obtaining employment, so as to further compress their employment space and ultimately reduce the economic participation rate of the chronically ill elderly. In terms of family participation, the chronically ill elderly have the highest level, and it is not significantly affected by the community home elderly care services, indicating that the social participation of the chronically ill elderly in China has a “home-based” role feature (36), that is, participation in family affairs is a rigid demand for the chronically ill elderly and is not easily met. From the perspective of voluntary participation and political participation, according to Maslow's hierarchy of needs theory, voluntary participation and political participation belong to social participation activities at a higher level of needs, which are often based on the basic premise that the survival and safety needs can be met. Therefore, the chronically ill elderly who use the community home elderly care services can reduce their worries and pressure on their own health care so as to devote more energy to voluntary and political activities.

When the chronically ill elderly are impacted by community home elderly care services, they will make differentiated social participation arrangements due to differences in their characteristics such as age, education, living arrangements, and community type constraints, resulting in group heterogeneity. This study finds that, with the increase in age, the influence of community home medical care services on the social participation of the elderly with chronic diseases will be differentiated due to the differences in age, education level, living arrangement, community type constraints, and other characteristics, resulting in group heterogeneity, which will gradually change from increasing voluntary participation and political participation to decreasing economic participation and family participation. This is because the phical function, psychological state, and social adaptability of the young elderly are at the best stage of the life cycle of the elderly. After using the community home elderly care services to meet their most basic needs for health care, there is still room to actively carry out voluntary participation and political participation at a higher level of demand. Therefore, the community home elderly care services mainly play a driving role in the social participation of the chronically ill elderly. For the chronically ill elderly whose abilities have degenerated in all aspects, the community home elderly care services are to play a convergent role in social participation. The chronically ill elderly mainly take the strategic approach of reducing economic participation and family participation. With the improvement of the education level of the chronically ill elderly, the impact of community home elderly care services on social participation has gradually shifted from reducing economic participation and increasing voluntary participation to increasing voluntary participation and political participation. In general, the chronically ill elderly who have a higher education level have more extensive economic and material resources or social support, so they have higher expectations of their own success and quality of life in their later years. After using community home elderly care services, they are more likely to carry out social activities such as voluntary participation and political participation with a higher demand level. In terms of home arrangement, the driving role of community home elderly care services for the voluntary participation and political participation of the chronically ill elderly who are not living alone is greater than that of the chronically ill elderly who live alone. At the same time, it also inhibits the economic participation of the chronically ill elderly who live alone. This is because the chronically ill elderly living alone are often the most vulnerable group among the elderly. Compared with the chronically ill elderly living alone, they lack support and care in life and spirit, and the quality of life is worrying. Therefore, community home elderly care services effectively make up for the lack of life care and spiritual comfort faced by the elderly living alone, so as to reduce the participation of subsistence society at the low demand level and increase the voluntary participation and political participation at the high demand level. In terms of community types, community home elderly care services have a driving effect on the voluntary participation and economic participation of the chronically ill elderly living in non-agricultural communities, but only on the voluntary participation of the chronically ill elderly in rural communities. This is because China's community home elderly care services have taken the lead in the development of urban communities, while the development of rural communities is relatively lagging and the level of community home elderly care services are relatively low, so the role of social participation of the chronically ill elderly is weak. In general, community home elderly care services have changed the social participation arrangements of the elderly with different characteristics of chronic diseases by driving and converging at the same time.

This study found that the community home elderly care services have an asymmetric impact on various social participation of the chronically ill elderly through the multidimensional health mediation mechanism, a negative impact on economic participation, family participation, and political participation through the physical health mediation mechanism, and a negative impact on economic participation and a positive drive on voluntary participation through the physical health mediation mechanism. Through the social health mediation mechanism, it positively drives economic and political participation and negatively drives family participation. In terms of physical health, physical health is the fundamental prerequisite for all social participation. Community home elderly care services negatively affect the social participation of the chronically ill elderly through the physical health mediation mechanism. On the one hand, it may be because the development of community home elderly care services in China is still at the initial stage, especially in the field of health care services, where there are shortfalls such as low resource investment, simple service, and low service quality. Therefore, it is difficult to improve the physical health of the chronically ill elderly, and may even delay their going to formal medical institutions for treatment; On the other hand, it may also be because a series of life care services provided by community home elderly care services indirectly deprive the chronically ill elderly of the opportunity to improve their physical health through self-care, which will negatively affect their physical health and thereby reduce their level of social participation. In the aspect of mental health, community home elderly care services such as door-to-door visits, service hotlines, and psychological counseling can reduce the psychological anxiety of the chronically ill elderly by improving their mental health, thereby reducing economic participation and creating subjective and objective conditions for the chronically ill elderly to participate in community voluntary activities. In terms of social health, community home elderly care services provide opportunities for the interaction between the chronically ill elderly and the social environment and provide social adaptability support for the chronically ill elderly to carry out economic and political participation.

This paper's policy enlightenment is: (1) With the continuous deepening of the interweaving between the growth of chronic diseases and the aging of the population in China, the development of a positive aging society must attach great importance to the important role of social participation for the chronically ill elderly. (2) Given that social participation arrangements for the chronically ill elderly face the combined effects of individual health factors, family factors, community factors, social structure factors and digital literacy factors, the process of stimulating social participation of the chronically ill elderly should not only focus on enhancing the development of their own physical, psychological and social adaptation health capabilities, but also scientifically guide the chronically ill elderly and society to combine individual factors, family factors, community factors, social structure factors and digital literacy factors, shape the awareness of social participation, reasonably assign social roles to the chronically ill elderly, and effectively balance the social responsibilities, rights and obligations of the chronically ill elderly. (3) Despite the rapid development of the community home elderly care services in China, it is still in the primary stage in general. Faced with small resource investment, simple service and low service quality, the outstanding weaknesses, how to achieve equity, fairness, sufficiency, sustainability and universal accessibility of the community home elderly care services should be the focus of the community home elderly care services policy incentives, especially the need to pay attention to the embedding of digital technology into community home elderly care services to enhance the overall capacity of China's community home elderly care services. (4) In view of the complex asymmetry of community home elderly care services affecting the social participation of the chronically ill elderly, when designing the combined menu of community home elderly care services, it is necessary to effectively grasp the organic match between the multiple health characteristics of the chronically ill elderly and the differences in social participation activities and pay special attention to the penetration, integration, and application of digital technology into the field of community home elderly care services, so as to effectively ensure the health of the chronically ill elderly, ensure that the elderly with different chronic diseases participate in social activities with high quality, enjoy a happy life with high quality, and promote the high-quality development of an aging society.

There are still some limitations in this study: it is difficult to explore the long-term dynamic impact of community home elderly care services on the social participation of the chronically ill elderly due to the selection of cross-sectional data for analysis. Since this study focuses on the three-dimensional mediation mechanisms of physical health, mental health, and social health of the chronically ill elderly, it is difficult to systematically grasp the complex mechanism of community home-based medical care services affecting social participation. In the digital era, digital technology has been fully integrated into all fields of the economy and society, and the smart elderly care service model has become increasingly popular. Digital participation has also become an important part of social participation. The relationship between community-smart elderly care services and the digital participation of the chronically ill elderly can be further studied.

## Data availability statement

The original contributions presented in the study are included in the article/supplementary material, further inquiries can be directed to the corresponding author.

## Author contributions

HJ: conceptualization, data curation, methodology, validation, resources, writing—original and editing, and screening questionnaires and tables. ZL: supervision, project management, writing—original and editing, preparation conceptualization, and formal analysis. All authors have read and agreed to the published version of the manuscript.
